# Factors Associated with Malaria Preventive Measures among Pregnant Women in Guinea

**DOI:** 10.1155/2021/9914424

**Published:** 2021-07-01

**Authors:** Abdourahamane Diallo, Almamy Amara Touré, Abdoulaye Doumbouya, Aboubacar Sidiki Magassouba, Falaye Traoré, Mamady Cissé, Ibrahima Barry, Ibrahima Conté, Diao Cissé, Abdourahim Cissé, Gnoume Camara, Alpha Oumar Bérété, Alsény Yarie Camara, Naby Yaya Conté, Abdoul Habib Beavogui

**Affiliations:** ^1^Centre Hospitalo-Universitaire Ignace Deen, Service de Gynécologie, Conakry, Guinea; ^2^Centre National de Formation et de Recherche en Santé Rurale de Mafèrinyah, Forécariah, Guinea; ^3^Department of Public Health, Faculty of Health Sciences and Techniques, Gamal Abdel Nasser University, Conakry, Guinea; ^4^Departement des Sciences Pharmaceutiques et Biologiques, Faculté des Sciences et Techniques de la Santé, Université Gamal Abdel Nasser de Conakry, Guinea; ^5^Direction Nationale Des Grandes Endemies et de la Lutte Contre la Maladie, Ministère de la santé, Conakry, Guinea; ^6^Sightsavers, Conakry, Guinea

## Abstract

**Background:**

Malaria control interventions have been scaled up, particularly those in pregnant women in Guinea. Despite that, coverage of key malaria preventive measure (MPM) indicators remains low. Therefore, it is vital to understand the reasons behind that, especially for the low coverage of sulfadoxine-pyrimethamine (SP) and long-lasting insecticide-treated bed nets (LLIN).

**Methods:**

We conducted a cross-sectional survey in nine district hospitals in Guinea. Pregnant women received for delivery were interviewed to collect sociodemographic and obstetrical parameters. Associated factors with MPMs were investigated through univariate analysis and classification and regression tree (CART).

**Results:**

A total of 2248 parturients participated in this study. Among pregnant women using mosquito nets (63.5% (61.4%, 65.5%)), only 41.2% (39.1%, 43.3%) had used it regularly during the last two weeks preceding delivery. Similarly, most pregnant women (57.9% (55.8%, 59.9%)) had received less than three doses of SP, and only a few pregnant women (23.9% (22.1%, 25.7%)) have benefited from full MPMs. Parturient's age, marital status, time spent in residence, place of residence, level of education, distance from home to the health centre, health conditions, occupation, head of the household's occupation, the presence of garbage and stagnant water in the neighbourhood, source of running water, and the number of pregnancies were significantly statistically associated with MPMs in pregnant women. However, the number of antenatal care visits (ANC), means of transportation used by the pregnant woman to accomplish ANCs, and stagnant water in the neighbourhood were the three preponderant factors.

**Conclusion:**

The low coverage of SP and LLINs among pregnant women requires revitalising some strategies, especially improving ANC coverage and more efforts to reduce inequalities in access to those services due to sociodemographic status. Education on the benefits of these MPMs should also be emphasised.

## 1. Introduction

Despite the progress made in the fight against malaria, pregnant women and children continue to suffer from its perverse effects [1]. According to the World Health Organisation's (WHO) malaria report in 2020, West Africa recorded 39% of malaria cases during pregnancy [1). Pregnant women are at high risk of malaria [2], and most of the time, they experience negative consequences [2–4]. This state of fact contrasts with the availability of adequate and approved prevention measures. For example, the administration of at least three doses of sulphadoxine-pyrimethamine (SP) during pregnancy is effective in reducing the rate of low birth weight [5, 6] and preventing anaemia [7]. In addition, the combined use of SP and long-lasting insecticide-treated bed net (LLIN) provides additional protection [8, 9]. To strengthen the implementation of scientific evidence that underpins malaria preventive approaches in pregnant women, WHO, since 2012, has recommended the use of SP during antenatal care (ANC) visits from the beginning of the second trimester of pregnancy until childbirth with an interval of one month between doses [10].

These recommendations were followed by Guinean health authorities and reinforced by educating pregnant women to adhere to malaria preventive measures. However, the 2018 demographic and health survey (DHS) showed a decrease in households owning mosquito nets from 47% to 43%.The proportion of pregnant women who slept under a mosquito net the night before the DHS remained constant at 28%, from 2012 to 2018 [11, 12]. Similarly, the proportion of pregnant women who received three or more SP doses increased from 23% to 36% in 2018 [12]. The latter findings are well below the expectations of the Guinean health authorities (80%). Moreover, this situation occurs in a context marked by the reinforcement of the Guinean health system after the 2014/2016 Ebola outbreak. In addition to this essential element, Guinea is a recipient of the President's Malaria Initiative (PMI) [13].

Thus, malaria prevention interventions have significantly increased. The availability of significant support in prevention should stimulate the good use of MPMs by pregnant women. In this context, it appears crucial to question reasons that hinder the achievement of these objectives. Therefore, this study identified the main sociodemographic and obstetrical factors that prevent the optimal use of MPMs in pregnant women in Guinea.

## 2. Methods

### 2.1. Study Sites

According to the general census, Guinea is a West African country located in the tropical region with an estimated population of 10,523,361 inhabitants in 2014 [14]. It is composed of four natural regions, and three of them were considered in this study. Lower Guinea is a coastal plain region covering 18% of the national territory and characterised by heavy rainfall varying from 3000 to 4000 mm of water per year. Upper Guinea is a region of plateaus and wooded savannahs covering 40% of the country's surface. The precipitation level varies between 1000 and 1500 mm of water per year with a hot and dry climate. Forest Guinea is made of mountainous massifs covering 20% of the national territory, characterised by a rainfall that varies between 2000 and 3000 mm per year with a humid climate. District hospitals serve as a second-level reference for health centres, which serve as a first level of reference for health posts. This study was conducted in nine (09) district hospitals, including Forécariah and Kindia in Lower Guinea, Kankan and Siguiri in Upper Guinea, Guéckedou and N'Zérokoréin Forest Guinea, and Matam, Kaloum, and Ratomain Conakry capital city.

### 2.2. Sample Size

This study analysed data from 2248 parturients and their newborns. The sample size was computed based on the formula *n* = *Z*^2∗^ (*P*^∗^ *Q*)/*i*^2^ where *n* is the desired sample size. Based on a study in Tanzania, the proportion of parturients with positive parasitaemia at childbirth was around 12% [15]. We opted for this sampling for two reasons:

1. Costing constraints: we have two variables of interest (SP and LLIN), which means we should have two different samples size
2. Choosing parasitemia percentage as a reference for calculating sample at delivery seems to be a reliable proxy to know the effectiveness of MPMs among pregnant women. The minimum sample size was 162 participants. However, if we consider the anticipated nonresponse rates, this size was increased from 162/0.80 = 202 participants and finally to 250 per district. Therefore, the number of pregnant women (2258) enrolled in this study was enough to draw a sound conclusion

### 2.3. Study Period and Data Collection

Parturients were enrolled as they went to health facilities for delivery. The study ran from 2 May to 24 September 2017. Data collectors received sufficient training about all study procedures, and collection tools were pretested to ensure their reliability. The interview was conducted in French for literate parturients and in local languages for illiterate ones.

### 2.4. Study Population

Only parturients with vaginal birth were included in this study.

### 2.5. Description of Study Factors

We first divided the factors into two categories:

(1-) Sociodemographic factors: age of the parturient (years), marital status (married, single), residence (the health district where she lives), duration of stay in that health district during pregnancy (less than six months, equal or more than six months), distance from residence to healthcentre (less than five kilometres, more than five kilometres), parturient's profession, occupation of the head of household, hygiene conditions in the parturient's neighbourhood (presence of stagnant water and/or garbage irregularly collected), use of traditional medicine (yes or no), household regimen (monogamy or polygamy), presence of an element of comfort (ventilator, air conditioner, or none), means of transportation to get to the prenatal consultation (walking, taxi, or personal car), characteristic of the residence (urban or rural), use of LLINs, regular use of LLINs during the last two weeks preceding childbirth (yes or no), and levels of education (none, primary, secondary, university)
(2-) Obstetrical factors: the number of SP doses, number of pregnancies, parity, the number of antenatal care visits (ANC), and use of other antimalarials during pregnancy

### 2.6. Ethical Considerations

The Institutional Review Board of the University of Conakry approved this study under number (0019/DM/CPM/17). Informed consent of all participants has been obtained. This study was conducted in compliance with Guinea ethical regulations.

### 2.7. Statistical Analyses

Qualitative variables were presented as proportions, quantitative variables as means, and confidence intervals calculated around the values. We recoded the following variables: age into three classes, adolescents 14-18 years old, young adults (19-35 years old), and mature (36-45 years old); the dose of SP according to the policy (less than 3doses and 3 or more doses); number of antenatal care visits (adequate when the number of ANC is 4 or more and not adequate when the number is less than 4); parity (primiparous = 1 childbirth, pauciparous = 2 − 3 childbirths, and multiparous ≥ 4 childbirths); and the number of pregnancies (primigravid = 1 pregnancy, paucigravid = 2-3 pregnancies, and multigravida ≥ 4 pregnancies). The variable of interest was constructed from two variables: the dose of SP and regular use of LLIN during the last two weeks preceding childbirth. The preventive measures were considered complete if pregnant women had received at least three doses of SP and had regularly used LLIN during the last two weeks preceding childbirth; otherwise, the preventive measures were considered incomplete (1) pregnant women who had received less than 3 doses of SP and had not regularly used LLIN, (2) pregnant women who had received less than three doses of SP and had used regularly used LLIN during the last two weeks preceding childbirth, and (3) pregnant women who had at least three doses of SP but had not regularly used LLIN during the last two weeks preceding childbirth). The regression tree was used to identify key factors associated with preventive measures. Analyses were performed using R software version 4.0.2 with the packages *compareGroups* and *ggparty*.

## 3. Results

**Figure 1:**
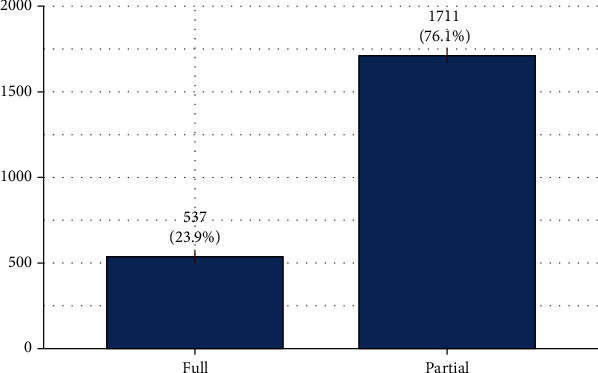
Malaria preventive measures (LLIN and SP).

Table 1 shows the description of the study population; we have enrolled 2248 participants. Young adults and married women were the most predominant with 80.6% (78.9%, 82.2%) and 83.9% (82.3%, 85.4%), respectively. Women who gave birth at term represented 72.2% (70.3%, 74.0%). The majority of women had lived more than six months in their residence, 89.6% (88.3%, 90.9%), and those living in urban areas were more represented 82.5% (80.8%, 84.0%). Most women's source of water was drilling 50.4% (48.3%, 52.4%). Pregnant women with no element of comfort were predominant 51.7% (49.6%, 53.8%), and pregnant women who had no formal education accounted for 47.5% (45.4%, 49.6%). Most pregnant women were housewives 56.7% (54.6%, 58.7%), and heads of households had mainly a liberal profession, 51.6% (49.6%, 53.7%). Most married women lived in a monogamous household, 56.7% (54.6%, 58.8%). Stagnant water and garbage were absent in most pregnant women's neighbourhood with 64.1% (62.0%, 66.0%) and 62.4% (60.3%, 64.4%), respectively. The majority of pregnant women went to the health centre by taxi, 34.8% (44.2%, 48.4%), and those living within a 5-kilometre radius from the health centre accounted for 79.1% (77.4%, 80.8%). Among pregnant women who used mosquito nets, 63.5% (61.4%, 65.5%), only 41.2% (39.1%, 43.3%) used them regularly during the last two weeks preceding childbirth. Primigravid pregnant women represented 36.6% (34.6%, 38.6%) and pauciparous 64.1% (62.0%, 66.0%). Most pregnant women received less than three SP doses, 57.9% (55.8%, 59.9%). Most of our participants did not use other antimalarial drugs or traditional medicine with 70.8% (68.9%, 72.7%) and 69.4% (67.4%, 71.3%), respectively. All variables were significantly associated with preventive measures in the univariate analysis except gestational age (weeks), parturient's occupation, gravidity, and parity (Table 2). Finally, only 23.9% of pregnant women benefited from full preventive measures (Figure 1).

**Figure 2:**
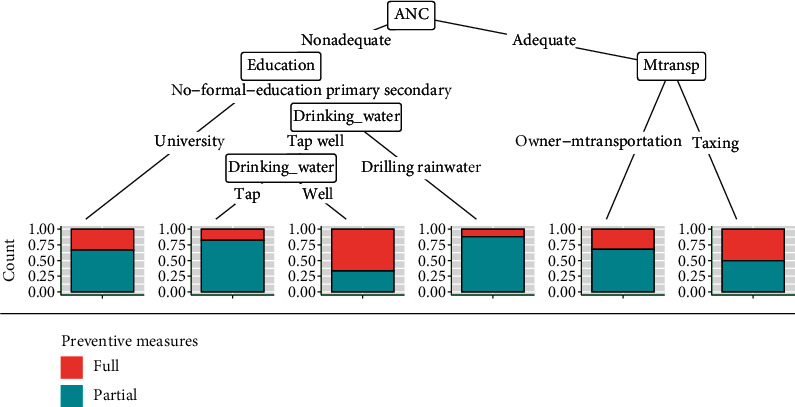
Regression tree of malaria preventive measures and sociodemographic and obstetrical characteristics. Mtransp: means of transportation.

The regression tree indicates that antenatal care visits (ANC) are the main factor that discriminates pregnant women over preventive measures. Based on adequate ANC, two groups stand out: pregnant women who had their means of transportation and women who walked or used public transportation to get to the health centre. On the other hand, with nonadequate ANC, two groups emerge according to the education (Figure 2):

1. Women who had reached university
2. Women who had either no formal education or primary or secondary level. Among those women, the source of drinking water plays a significant role

## 4. Discussion

Despite substantial support for malaria control, complete malaria preventive measures during pregnancy remain very limited in Guinea. Our analysis reveals several factors associated with the use of preventive malaria measures. Adolescents under 18 and women over 35 use preventive measures inadequately. The first group represents those with early pregnancy and probably single, as very few unmarried women have used preventive measures adequately. The second group of women constitutes the multigravida who, after several pregnancies, tend to neglect ANCs or other elements not clarified in this study, demotivating them to use preventive measures. Women who completed malaria preventive measures were mostly at term, thus confirming the value of these preventive measures [16, 17]. The duration of stay in the health district also appeared to be a factor influencing the adequate use of preventive measures; the longer a pregnant woman lives in a location, more optimal is the use of MPMs, which may imply that pregnant women living in a place for a short period are probably less inclined to observe preventive measures. Other sociodemographic and economic factors such as living in rural areas, the use of other water sources except for the tap, the distance from the house to the health centre, living in a polygamous regime, the head of the household, owning an element of comforts such as a fan or air conditioner, levels of education, and the use of traditional medicines during pregnancy are often associated with low living households in rural areas [18–21].

Regarding education level, our results indicate a more or less balanced coverage between women who had no level of education and those who reached secondary school. On the other side, it is difficult, based on this result, to confirm that the adequate use of preventive measures depends on the level of education. Although that is not confirmed in our study, it is a known fact that an adequate level of education is generally associated with fair use of healthcare services. One reason to explain the low coverage of MPMs in our study is the study setting, represented by district hospitals located in urban areas. As such, some women may opt for comfort items instead of specific preventive measures, such as using a fan or an air conditioner instead of mosquito nets.

Our hypothesis of the influence of socioeconomic level on adequate preventive measures can be based on another crucial factor: transportation to accomplish ANC. Pregnant women who had their cars had a lesser coverage of MPMs than those who did not. The previous findings reinforce our hypothesis regarding house comfort (fan or circulator) instead of using nets. We also found out that most pregnant women with full use of MPMs used drilling and tap as a drinking water source. The regression tree (CART) shows another picture of MPMs; we notice that women who had their drinking water from a well tend to use complete MPMs, but few of them (2.53%) had used full MPMs. As a result, poor socioeconomic conditions worsen inequalities in the use of MPMs. In addition, we observed more logically that these factors possibly caused the nonperformance of ANC, which is a key factor of full MPMs. The majority of our participants had not completed the minimum of four ANC visits recommended by the national policy for malaria prevention during pregnancy in Guinea. The added value of performing ANCs and the factors that determine it have been widely reported [22–27]. Several actions facilitate an increase in ANC coverage. For example, the Guinean ministry of health has adopted strategies that are not yet fully operational, such as (1) the “advanced strategy”, consisting of planning ANCs in remote areas and those beyond a 5-kilometre radius and (2) the “active research” to catch up with pregnant women who missed some ANC visits; this is also the use of ANC appointment reminders [28]. Poor implementation of these strategies and poor socioeconomic and demographic conditions explain the insufficient use of preventive measures.

The cross-sectional pattern of our findings does not allow us to draw a causal inference, and the factors analysed here are, for the most part, exogenous to malaria but may nevertheless have an indirect influence. A more rational way would have been to study the plausibility of the factors through a socioanthropological survey. Hospitals in urban areas are not necessarily the best choice, as many deliveries take place at home in Guinea [12], which could induce a selection bias. Guinea's four natural regions (Middle Guinea) were not part of this study due to logistics constraints. These limit, to a certain extent, our ability to generalise the results. Despite all of these limitations, the results of this study can guide the policy.

## 5. Conclusion

This study reveals that several sociodemographic (age, marital status, time spent in residence, place of residence, and level of education) and obstetrical (pregnancy and dose of SP) factors are associated with malaria preventive measures for pregnant women. Above all, it highlights the interest in the fair use of ANC. Policymakers should revitalise the education of pregnant women for the proper use of SP and LLINs. In the presence of significant support for malaria control, strengthening ANC strategies should be pursued and improved. It is crucial to find better strategies to avoid that sociodemographic conditions prevent vulnerable pregnant women from having full access to antenatal care services.

## Figures and Tables

**Table 1:** Description of the sample.

Study variables	Percentage	CI 95%	*N*
Age (years)			2248
14-18	14.5%	(13.1%, 16.1%)
19-35	80.6%	(78.9%, 82.2%)
35-45	4.89%	(4.04%, 5.87%)
Matrimonial status			2248
Single	16.1%	(14.6%, 17.7%)
Married	83.9%	(82.3%, 85.4%)
Gestational age			2248
Normal	72.2%	(70.3%, 74.0%)
Preterm	27.8%	(26.0%, 29.7%)
The duration of stay			2248
<6 months	10.4%	(9.13%, 11.7%)
≥6 months	89.6%	(88.3%, 90.9%)
Residence characteristic			2248
Urban	82.5%	(80.8%, 84.0%)
Rural	17.5%	(16.0%, 19.2%)
Source of drinking water			2248
Tap	36.5%	(34.5%, 38.5%)
Well	1.29%	(0.87%, 1.85%)
Drilling	50.4%	(48.3%, 52.4%)
Rainwater	11.9%	(10.6%, 13.3%)
Element of comfort			2248
Air conditioner	5.43%	(4.53%, 6.45%)
Fan	42.9%	(40.8%, 45.0%)
None	51.7%	(49.6%, 53.8%)
Levels of education			2248
No formal education	47.5%	(45.4%, 49.6%)
Primary school	13.8%	(12.4%, 15.3%)
Secondary school	27.2%	(25.3%, 29.1%)
University	11.5%	(10.2%, 12.9%)
Parturient's occupation			2248
Housewife	56.7%	(54.6%, 58.7%)
Freelance	31.2%	(29.3%, 33.1%)
Civil servant	12.1%	(10.8%, 13.6%)
Head of the household occupation			2248
Civil servant	26.3%	(24.5%, 28.2%)
Farmer	18.3%	(16.7%, 20.0%)
Freelance	51.6%	(49.6%, 53.7%)
Unemployed	3.69%	(2.95%, 4.56%)
Household regimen			2248
Monogamous	56.7%	(54.6%, 58.8%)
Polygamous	26.9%	(25.1%, 28.8%)
Single	16.4%	(14.9%, 18.0%)
Stagnant water			2248
No	64.1%	(62.0%, 66.0%)
Yes	35.9%	(34.0%, 38.0%)
Garbage			2248
Yes	37.6%	(35.6%, 39.7%)
No	62.4%	(60.3%, 64.4%)
Means of transportation			2248
Personal car	18.9%	(17.3%, 20.5%)
Walking	34.8%	(32.9%, 36.8%)
Taxi	46.3%	(44.2%, 48.4%)
Distance home-health centre (5 km radius)			2248
Yes	79.1%	(77.4%, 80.8%)
No	20.9%	(19.2%, 22.6%)
LLINs			2248
Yes	63.5%	(61.4%, 65.5%)
No	36.5%	(34.5%, 38.6%)
Regular use of LLINs			2248
Yes	41.2%	(39.1%, 43.3%)
No	58.8%	(56.7%, 60.9%)
Antenatal care visits			2248
Adequate	31.9%	(30.0%, 33.9%)	
Inadequate	68.1%	(66.1%, 70.0%)	
Gravidity			2248
Primigravid	36.6%	(34.6%, 38.6%)	
Paucigravid	38.3%	(36.3%, 40.4%)	
Multigravid	25.0%	(23.3%, 26.9%)	
Parity			2248
Primparous	0.53%	(0.28%, 0.93%)	
Pauciparous	64.1%	(62.0%, 66.0%)	
Multiparous	35.4%	(33.4%, 37.4%)	
SP doses			2248
<3 doses	57.9%	(55.8%, 59.9%)	
≥3 doses	42.1%	(40.1%, 44.2%)	
Use of other antimalarial drugs			2248
Yes	29.2%	(27.3%, 31.1%)
No	70.8%	(68.9%, 72.7%)
Use of traditional medicine			2248
Yes	30.6%	(28.7%, 32.6%)
No	69.4%	(67.4%, 71.3%)

**Table 2:** Association of preventive measures and sociodemographic and obstetrical characteristics.

Factors		Malaria preventive measures			*P* value
		Full		Partial	
		*N* = 537		*N* = 1711	
Age (years)					0.028
14-18	11.2%	(8.64%, 14.1%)	15.6%	(13.9%, 17.4%)
19-35	83.1%	(79.6%, 86.1%)	79.8%	(77.8%, 81.7%)
36-45	5.77%	(3.96%, 8.09%)	4.62%	(3.67%, 5.72%)
Matrimonial status					<0.001
Single	8.75%	(6.50%, 11.5%)	18.4%	(16.6%, 20.3%)
Married	91.2%	(88.5%, 93.5%)	81.6%	(79.7%, 83.4%)
Gestational age (weeks)					0.128
Normal	74.9%	(71.0%, 78.5%)	71.4%	(69.2%, 73.5%)
Preterm	25.1%	(21.5%, 29.0%)	28.6%	(26.5%, 30.8%)
The duration of stay					<0.001
<6 months	5.21%	(3.49%, 7.45%)	12.0%	(10.5%, 13.6%)
≥6 months	94.8%	(92.6%, 96.5%)	88.0%	(86.4%, 89.5%)
Residence					<0.001
Urban	92.7%	(90.2%, 94.8%)	79.3%	(77.3%, 81.2%)
Rural	7.26%	(5.22%, 9.79%)	20.7%	(18.8%, 22.7%)
Source of drinking water					<0.001
Tap	41.5%	(37.3%, 45.8%)	34.9%	(32.6%, 37.2%)
Well	2.98%	(1.71%, 4.79%)	0.76%	(0.41%, 1.30%)
Drilling	46.4%	(42.1%, 50.7%)	51.6%	(49.2%, 54.0%)
Rainwater	9.12%	(6.83%, 11.9%)	12.7%	(11.2%, 14.4%)
Element of comfort					<0.001
Air conditioner	11.4%	(8.80%, 14.4%)	3.57%	(2.74%, 4.56%)
Fan	56.2%	(51.9%, 60.5%)	38.7%	(36.4%, 41.0%)
None	32.4%	(28.5%, 36.5%)	57.7%	(55.4%, 60.1%)
Levels of education					<0.001
No formal education	18.1%	(14.9%, 21.6%)	9.47%	(8.12%, 11.0%)
Primary school	37.8%	(33.7%, 42.1%)	50.6%	(48.2%, 53.0%)
Secondary school	11.9%	(9.30%, 15.0%)	14.4%	(12.7%, 16.1%)
University	32.2%	(28.3%, 36.4%)	25.6%	(23.5%, 27.7%)
Parturient's occupation					0.085
Housewife	52.7%	(48.4%, 57.0%)	57.9%	(55.5%, 60.3%)
Freelance	33.3%	(29.4%, 37.5%)	30.5%	(28.3%, 32.8%)
Civil servant	14.0%	[(11.1%, 17.2%)	11.6%	(10.1%, 13.2%)
Head of the household occupation					<0.001
Civil servant	25.3%	(21.7%, 29.2%)	26.7%	(24.6%, 28.8%)
Farmer	11.7%	(9.13%, 14.8%)	20.4%	(18.5%, 22.4%)
Freelance	59.8%	(55.5%, 64.0%)	49.1%	(46.7%, 51.5%)
Unemployed	3.17%	(1.85%, 5.02%)	3.86%	(3.00%, 4.88%)
Couple regimen					<0.001
Monogamous	66.1%	(61.9%, 70.1%)	53.8%	(51.4%, 56.2%)
Polygamous	25.0%	(21.3%, 28.8%)	27.5%	(25.4%, 29.7%)
Single	8.94%	(6.66%, 11.7%)	18.7%	(16.9%, 20.6%)
Stagnant water					0.057
No	60.5%	(56.2%, 64.7%)	65.2%	(62.9%, 67.4%)
Yes	39.5%	(35.3%, 43.8%)	34.8%	(32.6%, 37.1%)
Rubbish					<0.001
Yes	30.4%	(26.5%, 34.4%)	39.9%	(37.6%, 42.3%)
No	69.6%	(65.6%, 73.5%)	60.1%	(57.7%, 62.4%)
Means of transportation					<0.001
Personal car	19.7%	(16.5%, 23.4%)	18.6%	(16.8%, 20.5%)
Walking	25.1%	(21.5%, 29.0%)	37.9%	(35.6%, 40.2%)
Taxi	55.1%	(50.8%, 59.4%)	43.5%	(41.2%, 45.9%)
Distance home-health centre (5 km radius)					0.004
Yes	74.7%	(70.8%, 78.3%)	80.5%	(78.6%, 82.4%)
No	25.3%	(21.7%, 29.2%)	19.5%	(17.6%, 21.4%)
ANC					<0.001
Adequate	45.4%	(41.2%, 49.8%)	75.2%	(73.1%, 77.2%)
Inadequate	54.6%	(50.2%, 58.8%)	24.8%	(22.8%, 26.9%)
Gravidity					0.062
Primigravid	33.7%	(29.7%, 37.9%)	37.5%	(35.2%, 39.9%)
Paucigravid	42.6%	(38.4%, 47.0%)	37.0%	(34.7%, 39.3%)
Multigravid	23.6%	(20.1%, 27.5%)	25.5%	(23.4%, 27.6%)
Parity					0.095
Primiparous	0.74%	(0.20%, 1.90%)	0.47%	(0.20%, 0.92%)
Pauciparous	67.4%	(63.3%, 71.4%)	63.0%	(60.7%, 65.3%)
Multiparous	31.8%	(27.9%, 36.0%)	36.5%	(34.2%, 38.9%)
Other antimalarial drugs					<0.001
Yes	22.3%	(18.9%, 26.1%)	31.3%	(29.1%, 33.6%)
No	77.7%	(73.9%, 81.1%)	68.7%	(66.4%, 70.9%)
Traditional medicine					<0.001
Yes	22.2%	(18.7%, 25.9%)	33.3%	(31.0%, 35.5%)	
No	77.8%	(74.1%, 81.3%)	66.7%	(64.5%, 69.0%)	

## Data Availability

The dataset supporting this article's conclusion is included in the article and its software R code.

## Supplementary Materials

Supplementary 1 Datasets.

Supplementary 2 R code.
